# Descriptive analysis of deaths associated with COVID-19 in Fiji, 15 April to 14 November 2021

**DOI:** 10.5365/wpsar.2022.13.4.964

**Published:** 2022-11-23

**Authors:** Nashika Sharma, Dashika Balak, Shaneel Prakash, Julia Maguire

**Affiliations:** a Ministry of Health and Medical Services, Suva, Fiji.; b Ministry of Lands and Mineral Resources, Suva, Fiji.; c World Health Organization Representative Office for the South Pacific, Suva, Fiji.

## Abstract

**Objective:**

There is limited published information about deaths due to coronavirus disease 2019 (COVID-19) in Fiji, the World Health Organization’s Western Pacific Region and low- and middle-income countries. This report descriptively analyses deaths directly associated with COVID-19 in Fiji by age group, sex, ethnicity, geographical location, vaccination status and place of death for the first 7 months of the 2021 community outbreak.

**Methods:**

A retrospective analysis was conducted of deaths directly associated with COVID-19 that occurred from 15 April to 14 November 2021 in Fiji. Death rates per 100 000 population were calculated by using divisional population estimates obtained from medical zone nurses in 2021.

**Results:**

A total of 1298 deaths relating to COVID-19 were reported, with 696 directly associated with COVID-19 and therefore included in the analysis. Of these, 71.1% (495) were reported from the Central Division, 54.6% (380) occurred among males, 75.6% (526) occurred among people of indigenous (iTaukei) ethnicity and 79.5% (553) occurred among people who were unvaccinated. Four deaths were classified as maternal deaths. The highest percentage of deaths occurred in those aged ^3^70 years (44.3%, 308), and the majority of deaths (56.6%, 394) occurred at home.

**Discussion:**

At-risk populations for COVID-19 mortality in Fiji include males, iTaukei peoples, and older (^3^70 years) and unvaccinated individuals. A high proportion of deaths occurred either at home or during the first 2 days of hospital admission, potentially indicating both a reluctance to seek medical care and a health-care system that was stressed during the peak of the outbreak.

Coronavirus disease 2019 (COVID-19) was first reported as clusters of unexplained pneumonia in late December 2019 in Wuhan, China, and was found to be caused by severe acute respiratory syndrome coronavirus 2 (SARS-CoV-2). The World Health Organization (WHO) declared COVID-19 a public health emergency of international concern on 30 January 2020. (*1*)

Fiji’s first COVID-19 case was imported on 15 March 2020 and resulted in a small local outbreak of 18 cases. Over the next year, 50 additional imported cases were reported without any community transmission. By 14 November 2020, 70 cases of confirmed COVID-19 had been reported, including two deaths. (*2*) There were no other cases until 15 April 2021, 364 days after the last reported, locally acquired case, when travellers tested positive for COVID-19 in government quarantine in a hotel. A subsequent locally acquired case of COVID-19 occurred when a hotel worker at the quarantine facility inadvertently had close contact with the infected travellers, and this marked the start of the second wave of the COVID-19 outbreak in Fiji. The sequencing of the local case’s specimen confirmed a SARS-CoV-2 Pango lineage B.1.617.2 variant – that is, the Delta variant – which at that time was classified as a variant of concern by WHO. (*1*)

During the same period in WHO’s Western Pacific Region, 10 of the 21 Pacific Island countries and territories reported cases of COVID-19: some had only imported cases contained in quarantine facilities (i.e. the Republic of the Marshall Islands, Samoa, the Solomon Islands and Vanuatu) and others had large-scale outbreaks (i.e. French Polynesia, Guam, New Caledonia, the Commonwealth of the Northern Mariana Islands, and Wallis and Futuna). The remaining 11 Pacific Island countries and territories remained COVID-free by closing their international borders and accepting only citizens and emergency support workers into their country or territory. (*1*)

There is limited published information regarding deaths due to COVID-19 in Fiji, the Western Pacific Region and low- and middle-income countries. (*3*-*5*) This report provides a descriptive analysis of the first 7 months of the 2021 outbreak for deaths directly associated with COVID-19 in Fiji by age group, sex, ethnicity, geographical location, vaccination status and place of death.

## Methods

We conducted a retrospective study of deaths directly associated with COVID-19 that occurred during the second wave of community transmission in Fiji between 15 April and 14 November 2021.

SARS-CoV-2 infection was identified using reverse transcription polymerase chain reaction (RT–PCR) testing. During this period, all RT–PCR samples were sent to the Fiji Centre for Communicable Disease Control, also known as Mataika House, which is Fiji’s national public health laboratory for analysis and reporting. Deaths were classified as either directly associated with COVID-19 (i.e. due to COVID-19) or indirectly associated with COVID-19 (i.e. people with COVID-19 infection at the time of death). (*6*) Classification was determined by the attending physician at the medical facility or by a mortality review panel, with COVID-19 categorized as a primary or secondary cause of death based on the case definition used by the Fiji Ministry of Health and Medical Services, (*7*) clinical records, medical history from relatives and results of COVID-19 investigations.

For each death directly associated with COVID-19, the Ministry of Health and Medical Services obtained the following information: age, sex, ethnicity, residential address, place of death, COVID-19 test type, date the specimen was collected for laboratory testing, date the specimen was tested, date of death, hospitalization status, date of hospital admission, COVID-19 vaccination status and dates of vaccination doses. In the analyses, deaths were classified as occurring at home or at a health facility (i.e. a health centre or hospital). Postmortem testing for COVID-19 was implemented for all deaths occurring during the study period.

A descriptive analysis was conducted for deaths directly associated with COVID-19. Death rates per  100 000 population were calculated by age group, sex, ethnicity and geographical location using division population estimates obtained from medical zone nurses in 2021. Note that the administrative boundaries (e.g. provincial boundaries) differ slightly from the medical division boundaries, hence medical zone demographic data were used.

## Results

**Fig. 1:**
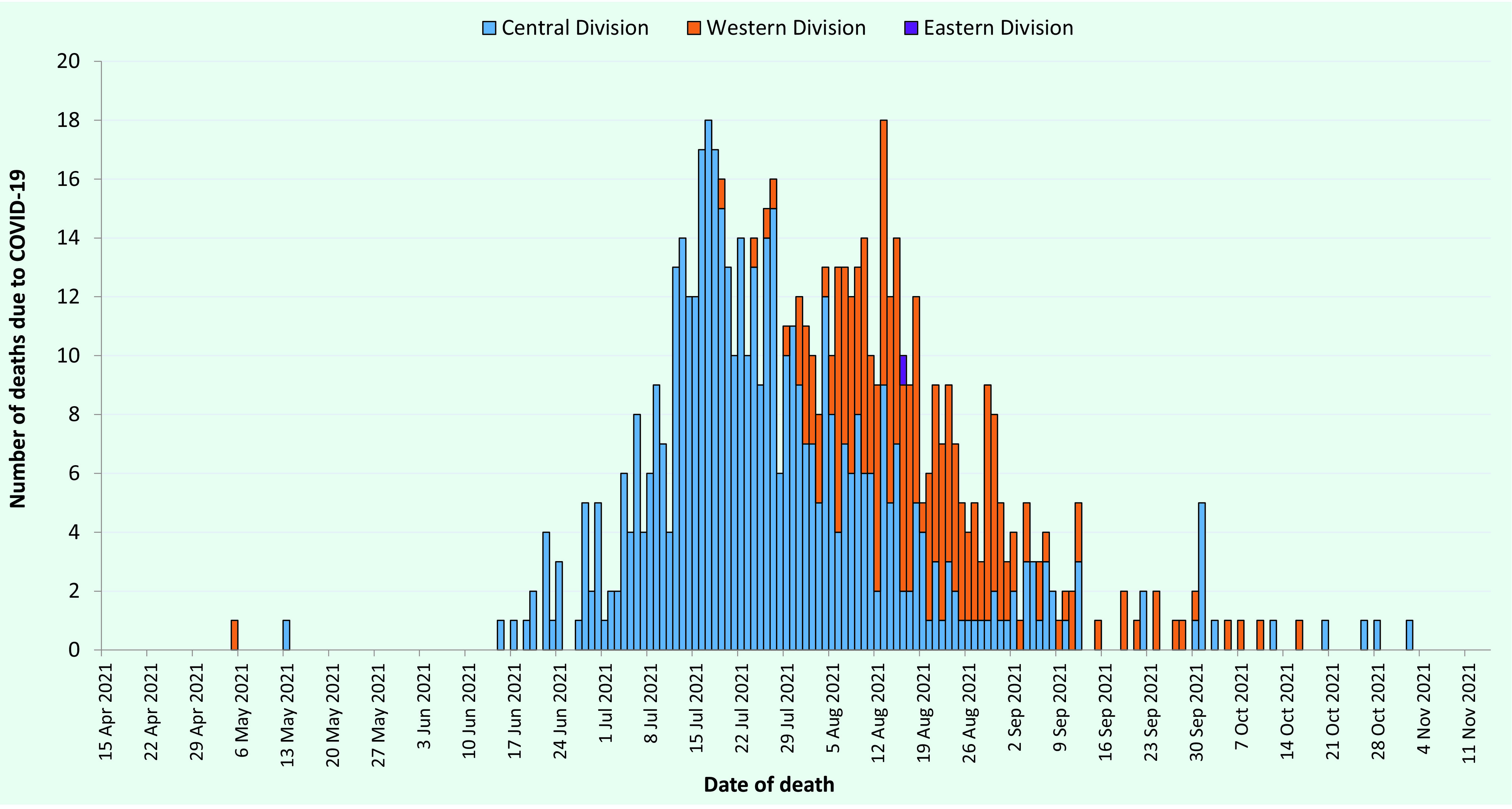
Deaths directly associated with COVID-19 by geographical division, Fiji, 15 April to 14 November 2021 (N = 696)

A total of 1298 deaths relating to COVID-19 were reported during the study period. Of these, 696 were categorized as being due to COVID-19 and were included in the analysis. For the first 4 months of the outbreak, deaths directly associated with COVID-19 primarily occurred in the Central Division; they later spread to the Western Division and then to the Eastern Division (**Fig. 1**).

[**insert** Figure 1]

Most deaths (71.1%, 495/696) were reported from the Central Division, and 54.6% (380/696) occurred among males, 75.6% (526/696) occurred among people of iTaukei ethnicity and 79.5% (553/696) occurred among unvaccinated people (**Table 1**). Although deaths were reported across all age groups, the median age of deaths due to COVID-19 was 67 years, and the highest percentage of deaths occurred in those aged (*3*)70 years (44.3%, 308/696). The death rate per age group–specific population increased with age (**Table 1**).

**Table 1:** Characteristics of 696 people whose death was directly associated with COVID-19, Fiji, 15 April to 14 November 2021

Characteristic	Deaths directly associated with COVID-19 (*n* = 696)	
	No. (%)	Rate/100 000 population
Sex		
Male	380 (54.6)	42.9
Female	316 (45.4)	35.7
Age (years)		
Median (IQR)	67.0 (21.0)	NA
Mean (SD)	65.6 (15.9)	NA
Age group		
< 20	9 (1.3)	2.7
20–29	9 (1.3)	6.3
30–39	22 (3.2)	16.4
40–49	58 (8.3)	56.3
50–59	120 (17.2)	132.4
60–69	170 (24.4)	327.3
^3^70	308 (44.3)	1079.2
Ethnicity		
iTaukei	526 (75.6)	NA
Fijian of Indian descent	139 (20.0)	NA
Other	31 (4.5)	NA
Place of death		
Hospital or health-care setting	302 (43.4)	NA
Home	394 (56.6)	NA
Vaccination status		
Unvaccinated	553 (79.5)	NA
One dose	130 (18.7)	NA
Two doses	13 (1.9)	NA
Geographical division		
Central	495 (71.1)	123.4
Western	200 (28.7)	56.3
Eastern	1 (0.1)	2.6
Northern	0 (0.0)	0

IQR: interquartile range; NA: not applicable; SD: standard deviation.

Four deaths were classified as maternal deaths, all of which occurred during the postpartum period at divisional hospitals between 4 and 6 days from the date of admission (data not shown). Three maternal deaths occurred in the Central Division, while one death occurred in the Western Division. The mean age of those categorized as a maternal death was 36.5 years (median, 35 years), and all women in this group were reported to be unvaccinated.

**Fig. 2:**
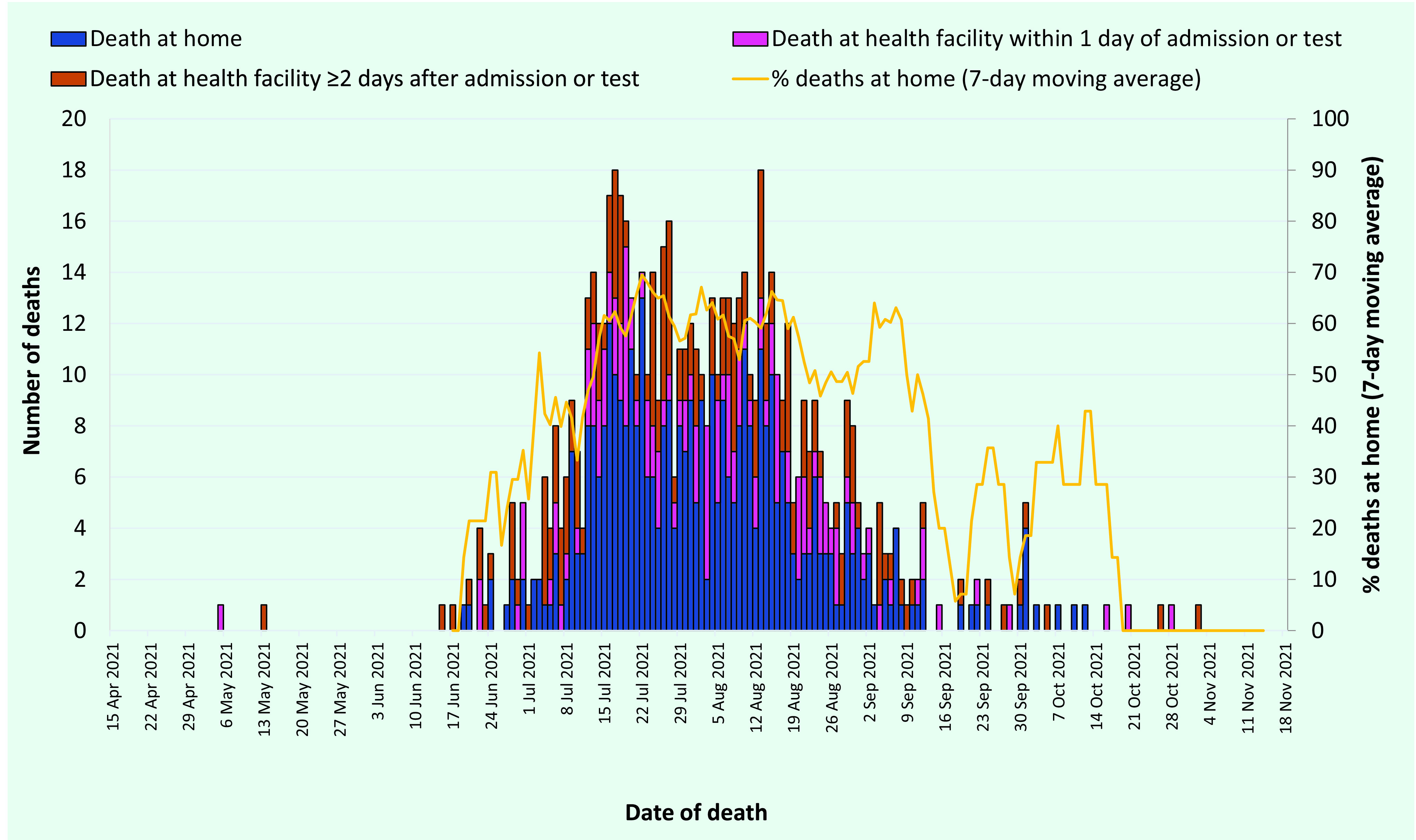
Deaths directly associated with COVID-19 by place and date of death, Fiji, 15 April to 14 November 2021 (N = 696)

The majority of deaths directly associated with COVID-19 occurred at home (56.6%, 394/696), and of these 53.8% (212/394) were among males, 86.3% (340/394) were among iTaukei people and 50% (197/394) were among people aged (*3*)70 years. Of the 43.4% (302/696) of deaths that occurred in a hospital or health-care setting, 56.3% (170/302) were among males, 61.6% (186/302) were among iTaukei people and 36.8% (111/302) were among people aged (*3*)70 years (**Table 2**). Of the deaths that occurred in the hospital or health-care setting, 44.0% (133/302) occurred within 1 day of admission, 9.3% (28/302) occurred 2 days after admission and 46.7% (141/302) occurred (*3*)3 days after admission. Early in the outbreak when there were fewer cases, less than half of deaths occurred at home; however, during the peak of the outbreak (15 July–12 August) more than 60% of deaths occurred at home rather than in a health facility (**Fig. 2**). From September onwards, this proportion decreased as the number of cases and deaths decreased.

[**insert** Figure 2]

**Table 2:** Characteristics of 696 people whose death was directly associated with COVID-19 by place of death, Fiji, 15 April to 14 November 2021

Characteristic	No. (%) of deaths	
	At home (*n* = 394)	In hospital or health-care setting (*n* = 302)
Sex		
Male	212 (53.8)	170 (56.3)
Female	182 (46.2)	132 (43.7)
Age group (years)		
< 20	3 (0.8)	6 (2.0)
20–29	3 (0.8)	6 (2.0)
30–39	10 (2.5)	12 (4.0)
40–49	22 (5.6)	36 (11.9)
50–59	61 (15.4)	59 (19.5)
60–69	98 (24.9)	72 (23.8)
^3^70	197 (50.0)	111 (36.8)
Ethnicity		
iTaukei	340 (86.3)	186 (61.6)
Fijian of Indian descent	39 (9.9)	100 (33.1)
Other	15 (3.8)	16 (5.3)

The place of deaths directly associated with COVID-19 (i.e. at home or at a health centre or hospital) varied by division. In the Central Division, most deaths occurred at home (60.2%, 298/495), with the remaining occurring in hospitals (35.2%, 174/495) and at health centres (4.6%, 23/495). Conversely, in the Western Division, most deaths occurred in hospitals (52.0%, 104/200), with slightly fewer occurring at home (47.5%, 95/200) and 0.5% (1/200) occurring at a health centre. In the Eastern Division, one death occurred at home; no deaths were reported in the Northern Division during the study period.

## Discussion

Our study describes deaths directly associated with COVID-19 occurring in Fiji during its second wave of community transmission in 2021. Most of these deaths occurred among males, people aged (*3*)70 years and those living in the Central Division (the most populous division in Fiji).

Geographically, the deaths directly associated with COVID-19 followed a similar pattern to that of the cases, occurring first in the Central Division, then the Western Division and later in the Eastern Division. (*8*) The delayed spread of cases through the country can be attributed to the restriction of movement across the major divisional borders and from areas with localized outbreaks. With cases initially concentrated in the Central Division, the remaining divisions had the opportunity to prepare their health systems for an influx of cases and also rapidly increase vaccination coverage to prevent widespread disease transmission.

In this study, more than half of the deaths directly associated with COVID-19 were among males, which is consistent with other studies, demonstrating that male sex is associated with higher mortality. (*9*, *10*) A paper by Nguyen et al. additionally reported that male sex is not only associated with a higher rate of mortality but also with a higher rate of respiratory intubation and a longer length of hospital stay. (*9*) Although there is limited information about the relationship between sex and COVID-19, the literature has highlighted the importance of understanding the role of comorbidities, immune system responses and sex hormones as drivers of COVID-19 mortality. (*9*, *10*)

During our study period, deaths directly associated with COVID-19 were reported in all age groups. However, the number and rate of COVID-19 deaths were highest in those aged (*3*)70 years, highlighting that COVID-19 mortality increases with age. (*11*-*13*) A paper by Jergović et al. emphasized that the loss of immune function and reduced protection from infectious agents that occur with age are factors associated with increased disease severity and mortality from COVID-19. (*12*)

The deaths directly associated with COVID-19 in our study population occurred predominantly among unvaccinated people, who accounted for 79.5% of deaths, whereas 18.7% of those who died had received one dose of vaccine and 1.9% had received two doses. This is similar to other studies, highlighting that mortality from COVID-19 is higher in the unvaccinated population than the vaccinated population. (*14*-*17*) COVID-19 vaccinations have successfully reduced the incidence and severity of, and hospitalization and deaths from, COVID-19. (*14*-*17*) Although many countries are using different vaccines and booster regimens, it is evident that COVID-19 vaccinations have the potential to reduce morbidity and mortality. (*15*-*17*) The Fiji national COVID-19 vaccination programme commenced on 6 April 2021, with 61 667 individuals aged > 18 (approximately 10% of the eligible population) receiving their first dose of vaccine by the end of April 2021; by 14 November 2021, 599 423 (97% of the eligible population) had received their first dose and 553 943 (89.6%) had received their second dose.

During the study period, four maternal deaths were reported. We have limited antenatal, intrapartum and postpartum information about these maternal deaths, so it is difficult to draw meaningful associations with other studies conducted around the world; however, a review of the literature highlights that pregnant women are at higher risk of severe COVID-19 infection; of needing Caesarean delivery, intensive care admission and invasive ventilation; and of having adverse maternal and neonatal outcomes. (*18*-*22*) Three separate studies conducted in the United States of America and Scotland found that severe complications known to be associated with COVID-19 in pregnancy (such as admission to a critical care unit, perinatal mortality and developing severe or critical COVID-19 infection) were more common in pregnant women who were unvaccinated at the time they were diagnosed with COVID-19 than in vaccinated pregnant women. (*23*-*25*) Therefore, this highlights the importance of vaccinating pregnant women to reduce the severe maternal and neonatal health outcomes associated with COVID-19.

We found that although all ethnicities in Fiji were at risk of contracting and dying from COVID-19, indigenous populations (i.e. iTaukei) had a disproportionately higher rate of death from the disease. A review of the literature shows that globally indigenous populations seem to have higher rates of infection, more severe disease, higher rates of hospitalization, and poorer health and health outcomes from COVID-19. (*26*-*28*) Although there is limited knowledge about the relationship between ethnicity and COVID-19 morbidity and mortality, research suggests that pre-existing social, economic, political and cultural determinants of health are important factors in the health and health outcomes of indigenous populations. (*26*) Therefore, it is important to collect timely, relevant, high-quality and disaggregated data to better understand the needs of vulnerable and at-risk populations and to ensure that COVID-19 response and mitigation measures are delivered in a way that ensures health equity and health equality. (*29*)

We found that the majority of deaths directly associated with COVID-19 occurred at home (56.6%). While the reason for this is not clear, it is important to understand a population’s health-seeking behaviours and the factors that drive these behaviours. Two studies conducted in Pakistan and Viet Nam examined health-seeking behaviours and factors that altered these during the COVID-19 pandemic. (*30*, *31*) They found that individuals increased self-medication with unprescribed drugs, decreased their hospital visits and had an increased preference for visiting private general practitioners, traditional healers and unregistered clinics rather than visiting government facilities. The main factors that limited or altered health-seeking behaviours, or both, during the pandemic included fears of being stigmatized, of whole families being transferred to quarantine facilities and of disclosing past activities to contact tracing teams, and these fears were enhanced by misinformation, panic and uncertainties that spread over social media platforms. (*30*, *31*) The two countries in these studies are developing countries, and these findings may be applicable to the context in Fiji. It is also important to consider the immense and unprecedented stress placed on the health-care system in Fiji during the peak of the outbreak, and its impact on the system’s ability to provide adequate and timely services to people with COVID-19. Indeed, we found that a high proportion of deaths occurred at home or soon after hospital admission, but this may be due to multiple factors, such as a limited ability to identify people with deteriorating health, limited availability of transportation to hospital and limited bed capacity to treat patients within the hospital, rather than a lack of health-seeking behaviour. As the outbreak progressed, strategies were implemented to increase the ability of the health system to identify those most at risk of severe disease and place them into an appropriate care pathway. More research on health-seeking behaviours and the factors that drive these behaviours within the context of Pacific Island countries and territories is pivotal for informing future pandemic response and mitigation measures. (*30*, *31*)

There were some limitations to this study. The classification of deaths depended on the assessment of the attending physician and, therefore, there was potential for misclassification. If the death occurred outside a health-care facility (e.g. at home), there may have been a delay in receiving the death certificate; therefore, there is potential for delayed reporting or underreporting of deaths during our study period. We were also unable to calculate mortality rates by ethnicity due to a lack of recent population data. In this study, deaths directly associated with COVID-19 were reported by geographical divisions; however, it would be valuable to analyse deaths by urban, periurban and rural settings because some communities have poorer access to health-care services, water, hygiene and sanitation and, as a result, are reported to have poorer health and health outcomes. It would also be interesting to assess the common signs and symptoms, and severity of COVID-19 disease, as well as underlying comorbidities, especially since about 80% of all deaths that occur in Fiji are due to noncommunicable diseases. (*32*) However, this information is not reported in the Medical Cause of Death Certificates, and a detailed review of inpatient data and clinical notes would be required. Occasionally, the clinical severity of disease was classified on the death certificate, but the investigative team was unsure how physicians classified the severity and whether all physicians in Fiji used standard definitions. Therefore, this information was not analysed. Understanding the common comorbidities among those who died from COVID-19 would help to highlight populations that are at risk for severe outcomes in Fiji, and understanding the severity of disease would provide a way to assess the required levels of health-care preparedness, health-care delivery and future clinical and public health forecasting in a developing country like Fiji. Information on the common signs and symptoms of COVID-19 experienced within our population can be used to increase knowledge and awareness among our frontline and allied health-care workers and can also be used to develop risk communication material to increase knowledge of and awareness about COVID-19 among our population.

In addition, understanding the patterns of disease among other at-risk populations would be useful, such as individuals with underlying mental health disorders or illnesses, those who are immunocompromised, those who have a disability, and residents of aged-care facilities, as well as low-income or unemployed individuals, unhoused individuals, and members of the lesbian, gay, bisexual, transgender and queer communities; however, this information was not available. Having these data would allow health promotion activities to be targeted to reduce morbidity and mortality in these groups.

This retrospective analysis of deaths directly associated with COVID-19 that occurred in Fiji during the second wave of the pandemic (15 April to 14 November 2021) found that at-risk groups included male, indigenous (iTaukei), older ((*3*)70 years) and unvaccinated individuals. Therefore, we conclude that individuals belonging to these risk groups in Fiji should adhere to the recommended COVID-19 precautions and preventive measures to avoid becoming infected with SARS-CoV-2, and we recommend that future public health prevention strategies, health promotion activities, risk communication materials and public health policies for COVID-19 in Fiji are tailored to these at-risk populations. Strategies should include providing education about the signs and symptoms of severe and progressing COVID-19, and increasing the capacity of health systems to identify and respond to a rapid influx of deteriorating patients.
